# Safety of treatment regimens for drug-resistant TB over a 15-year period: a scoping review

**DOI:** 10.5588/ijtldopen.25.0728

**Published:** 2026-04-13

**Authors:** M. Amiri, M. Cheraghi, M.J. Nasiri, D.R. Silva, G. Sotgiu, L. D’Ambrosio, R. Centis, I. Kontsevaya, H.K. Gandhi, D. Oberdhan, V. Girbinger, M. Dara

**Affiliations:** 1 Department of Microbiology, School of Medicine, Shahid Beheshti University of Medical Sciences, Tehran, Iran;; 2 Faculdade de Medicina, Universidade Federal do Rio Grande do Sul (UFRGS), Porto Alegre, Brazil;; 3 Clinical Epidemiology and Medical Statistics Unit, Department of Medicine, Surgery and Pharmacy, University of Sassari, Sassari, Italy;; 4 Public Health Consulting Group, Lugano, Switzerland;; 5 Servizio di Epidemiologia Clinica delle Malattie Respiratorie, Istituti Clinici Scientifici Maugeri, IRCCS, Tradate, Italy;; 6 Otsuka Novel Products GmbH, Munich, Germany;; 7 Otsuka Pharmaceutical Development & Commercialization, Inc., Princeton, NJ, USA.

**Keywords:** tuberculosis, MDR-TB, DR-TB, meta-analysis, tolerability, adverse events

## Abstract

**BACKGROUND:**

A previous review summarised the evolution and efficacy of the regimens to treat rifampicin-resistant/multidrug-resistant TB (RR/MDR-TB), underscoring the persistent need for efficacious shorter treatments. The aim of this scoping review was to explore safety, quality of life (QoL), and unmet needs associated with RR/MDR-TB in studies published between 2009 and 2024.

**METHODS:**

We searched PubMed/MEDLINE, Embase, Cochrane CENTRAL, Scopus, and Web of Science for studies reporting safety, QoL, and unmet needs in the last 15 years.

**RESULTS:**

Fifty-seven studies including 9,874 patients were identified, with significant variation in geographic distribution, sample size, and other core variables. The overall proportion of serious adverse events (AEs) ranged between 0.2% and 10.1% in retrospective studies, 1.0%–72.4% in prospective cohorts, and 20.0%–25.0% in experimental studies, with no data on QoL. Almost all studies containing linezolid (LZD) reported gastrointestinal and haematological AEs. In studies based on individual patient data, AEs associated with bedaquiline (1.7%–2.4%) and fluoroquinolones (3%–4%) were less frequent than those associated with LZD (14.1%–17.2%). The World Health Organization 95% credible interval range was 10.1%–27.0%.

**CONCLUSION:**

While efficacious RR/MDR-TB regimens are recommended, individual drugs still cause AEs potentially leading to decreased adherence. New efficacious treatments with improved safety/tolerability profiles are needed.

With 10.7 million newly detected TB cases, and 1.23 million deaths as reported by the World Health Organization (WHO), TB continues to be a global clinical and public health priority. WHO estimated that, in 2024, about 390,000 people developed multidrug-resistant (MDR)/rifampicin-resistant (RR)-TB.^1^ Though the clinical and programmatic management of drug-susceptible (DS)-TB is easier, the spread of MDR-TB, pre-extensively drug-resistant (pre-XDR), and extensively drug-resistant (XDR)-TB pose additional challenges.^2^ In addition to the complexity of providing a rapid and accurate diagnosis, different regimens are needed for the treatment of DR-TB cases. These include new and repurposed drugs, which are associated with more frequent and often severe adverse events (AEs).^3^ WHO classifies drugs used in the treatment of RR/MDR-TB as: Group A (highly effective drugs like fluoroquinolones [FQs] [levofloxacin, moxifloxacin], bedaquiline [BDQ], and linezolid [LZD], which should be included in a regimen unless there is a contraindication), Group B (effective drugs like clofazimine and cycloserine/terizidone that are recommended as second-choice additions), and Group C (drugs that have a lower efficacy, a worse balance of benefit to harm, or are poorly tolerated, such as pyrazinamide, ethambutol, amikacin, and streptomycin. These are used when Group A and B drugs are not available or tolerated). Although the other WHO Group A drugs (FQs and BDQ) are well tolerated, LZD has demonstrated increased toxicity, as well as some of the drugs belonging to Groups B and C.^2–4^

The residual unmet need for efficacious treatments with shorter durations for RR/MDR-TB was documented in a recent systematic review and meta-analysis^5^ describing the evolution of the regimens to treat RR/MDR-TB between 2009 and 2024. The study reviewed the efficacy of the regimens developed in the past decades to treat DR-TB with focus on new drugs (BDQ, delamanid [DLM], and pretomanid [Pa]) and shorter regimens.^2,6,7^ However, efficacy is only one component of treatments for RR/MDR-TB. Safety, tolerability, and associated quality of life (QoL) can impact adherence to regimens and patient outcomes. While extensive literature on AEs related to RR/MDR-TB treatment exists,^3,8,9^ a comprehensive review and meta-analysis summarising findings related to the tolerability of DR-TB regimens is needed.

The aim of the present scoping review is to explore additional aspects of the new regimens, such as safety and QoL reported between 2009 and 2024, as well as associated unmet needs.

## METHODS

We conducted a scoping review using a systematic approach to identify relevant literature from databases including PubMed/MEDLINE, Embase, Cochrane CENTRAL, Scopus, and Web of Science. Search terms comprised combinations of keywords related to TB, drug resistance, treatment outcomes, safety, tolerability, AEs, and QoL. Studies published between 1 January 2009 and 1 June 2024 were included to ensure a comprehensive review of recent developments. AEs encompass any problematic medical manifestation after exposure to a medicine, which is not necessarily caused by that medicine.^10^

### Study selection criteria

We included observational and experimental studies reporting on the safety of new treatment regimens for DR-TB. This encompassed reported AEs, treatment tolerability, and impacts on QoL. Controlled trials (both randomised and non-randomised) as well as observational studies (including cohort, case-control, and cross-sectional designs) were considered to capture a broad spectrum of evidence on DR-TB treatment safety and tolerability. Case reports/series, reviews, editorials, or conference abstracts were excluded. Studies focusing exclusively on children (<14 years of age), adolescents, or pregnant women were also excluded due to potential differences in treatment outcomes and adverse effect profiles compared to adult and non-pregnant populations. In addition, to take advantage of global information, a descriptive review was conducted, capturing the available information from existing studies based on individual data.^3^

### Review process

All identified articles were uploaded to EndNote, and duplicates were removed manually. Two reviewers (MA, MCH) independently screened titles and abstracts of the articles. Any disagreements were resolved by a third reviewer (MJN). Extraction tables were reviewed randomly by two other co-authors (IK and MD), and subsequently, the team evaluated full texts of all potentially eligible studies, and any disagreements were resolved by the third reviewer.

### Data extraction

Two reviewers (MA and MCH) systematically extracted data into a predefined spreadsheet using Microsoft Excel. In case of any discrepancies, a third reviewer (MJN) was involved. This process involved compiling information on various AEs reported across the studies, including QTcF prolongation (QT interval, corrected for heart rate according to the Fridericia formula, >500 ms), hepatic disorders/elevated liver enzymes, renal failure/increased creatinine levels, optic neuropathy/blurred vision, ototoxicity/hearing loss, haematological disorders (anaemia, thrombocytopenia, eosinophilia), gastrointestinal (GI) symptoms (diarrhoea, vomiting, nausea, abdominal pain, pancreatitis), peripheral neuropathy, electrolyte disturbances, arthralgia, psychiatric disorders, dermatologic symptoms, weakness, cardiac disorders (palpitations), nervous system disorders (memory loss, seizure), musculoskeletal disorders (pain, atrophy), hypothyroidism, and fever.

Furthermore, the studies were analysed to identify AEs attributable to specific drug(s).

Serious adverse events (SAEs) as per WHO definition^3^ included death or a life-threatening event, hospitalisation or prolongation of hospitalisation, persistent or significant disability, or congenital anomaly. SAEs included grade 3–5 AEs (grade 3: serious; grade 4: life-threatening; grade 5: death).

### Comparative analysis

As part of the descriptive review, we conducted a comparative analysis of AEs associated with DR-TB treatments described in the selected studies and estimates from the latest WHO DR-TB guidelines.^11^

### Ethical statement

As a scoping review, ethical approval was not required.

## RESULTS

The Figure illustrates the systematic process of study selection for inclusion in the scoping review. Initially, a comprehensive search yielded a total of 9,875 articles across various databases and sources. After removing duplicates, 5,235 unique records were screened based on their titles and abstracts. Following this initial screening, 590 articles underwent full-text assessment for eligibility criteria. Ultimately, 57 studies met the inclusion criteria and were included in the scoping review for detailed analysis.

**Figure.**
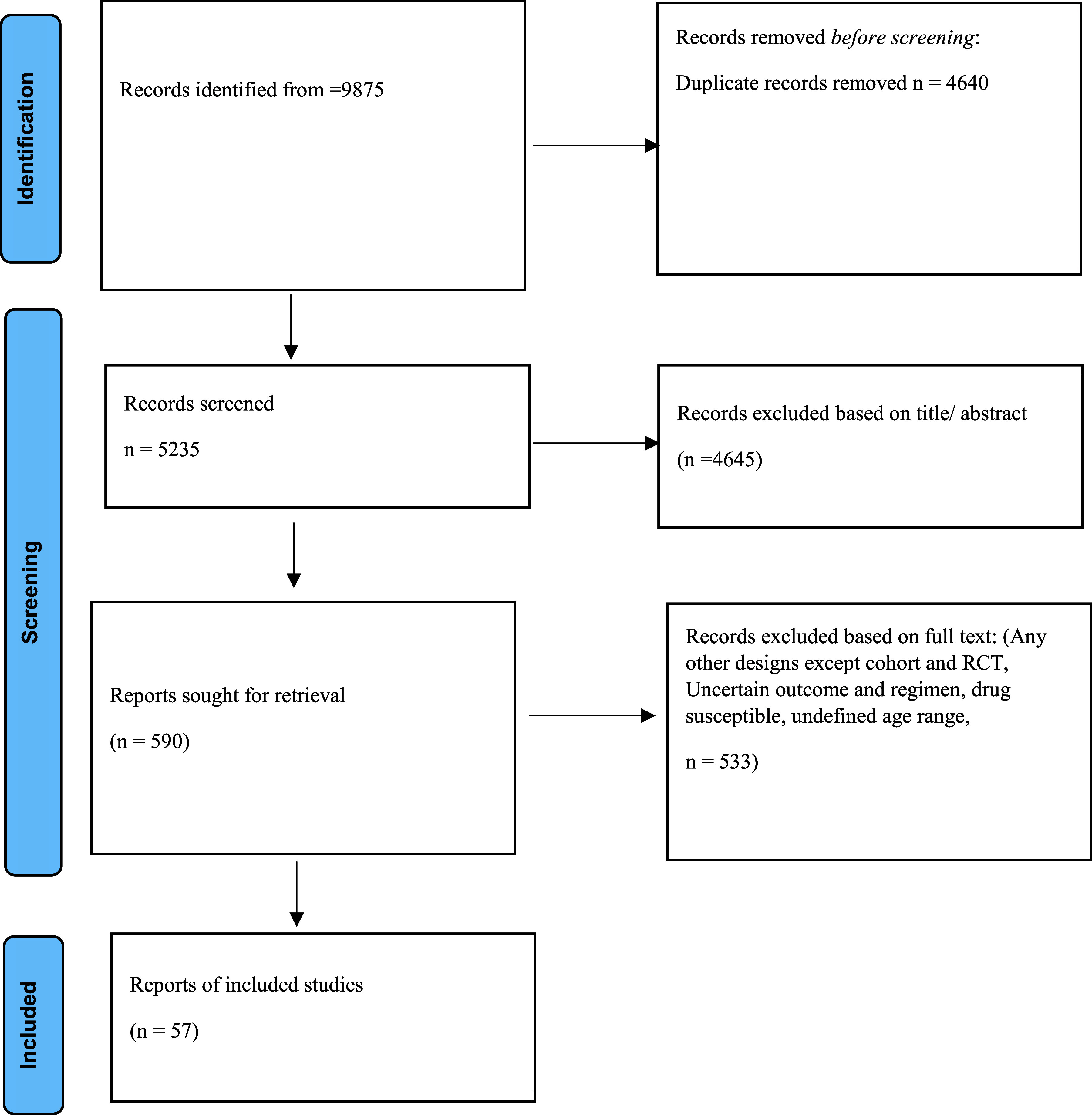
Flow chart of study selection for inclusion in the scoping review.

### Study characteristics

The characteristics of the included studies are summarised in Supplementary Data Table S1, encompassing 57 studies^12–68^ with a total of 9,874 patients. The studies varied in design: 46 (80.70%) were observational studies, while 11 (19.29%) were randomised controlled trials. As some studies included more than one treatment arm with different regimen or duration as well as different demographic information, we analysed them separately. In total, 82 studies/arms were included in our review comprising 58 observational studies/arms (35 retrospective and 23 prospective) and 24 randomised controlled studies/arms. These studies were conducted in various countries, reflecting a broad geographic distribution: 10 studies (17.54%) in South Africa involving 1,917 patients (19.41%); 10 studies (17.54%) in India with 1,131 patients (11.45%); 10 studies (17.54%) in China with 1,928 patients (19.52%); and 7 studies (12.28%) in South Korea involving 1,501 patients (15.20%), as well as Armenia, Austria, Belarus, Canada, Congo, Eswatini, Ethiopia, France, Georgia, Nepal, Sweden, Taiwan, Tanzania, and Vietnam among others. The number of patients ranged from as few as 14^51^ to as many as 741^48^ with a median cohort size of 120 patients. Treatment regimens varied widely, often involving combinations of medications tailored to specific DR-TB types, commonly including BDQ, clofazimine, levofloxacin, LZD, and moxifloxacin. The mean age of participants was reported in 51 studies (89.47%) and ranged from 24 to 58.8 years across studies, with an overall mean age of 38 years. Gender distribution showed a predominance of male patients, with 54 studies (94.73%) reporting details on this; HIV status was variably reported, with 47 studies (82.45%) indicating HIV-positive patients while several studies reported no HIV-positive cases. Body mass index (BMI; kg/m^2^) was reported in 29 studies (50.88%), with a range between 16.48^47^ and 22.00.^23^ Diabetes Mellitus status was reported in 34 studies (59.65%), with a small proportion of patients affected. Smoking status was reported in 18 studies (31.57%), with a range between 10.81%^43^ and 73.33%,^15^ indicating variable prevalence. The types of DR-TB covered included MDR-TB in 42 studies (73.68%), XDR-TB in 28 studies (49.12%), pre-XDR-TB in 20 studies (35.08%), and RR-TB in 14 studies (24.56%). No relevant information on QoL was found in the studies analysed. Treatment duration ranged from 6 to 48 months.

Six (10.34%) of 58 observational (e.g., non-randomised clinical trials) studies or arms (Supplementary Data Table S1) provided no details on treatment duration. Of the remaining 52 studies/arms, 17 (32.69%) evaluated shorter regimens (4 evaluating regimens of 6 months and 13 regimens from 6 to 12 months) and 35 (67.30%) longer regimens. Following the WHO-recommended longer regimens in 2016, the data showed a strong concentration of studies around the 18- to 24-month duration. Due to the heterogeneity among studies, it was not possible to describe specific safety trends related to regimen use, composition of the regimens, and treatment duration.

### Total adverse events

Supplementary Data Table S2 summarises the total AEs observed in the included studies, comprising a total of 9,874 patients. In observational studies, which collectively included 7,604 patients, the incidence of SAEs varied notably. Among retrospective cohort studies encompassing 5,158 patients, SAEs ranged from 0.24%^23^ to 10.11%.^18^ Prospective cohort studies involving 2,446 patients documented SAE rates ranging from 1.05%^26^ to 72.41%.^36^ Experimental studies/arms, involving 2,270 patients, reported SAE rates ranging from 20.00% to 25.00%.^58^

### Types of adverse events

Supplementary Data Table S3 provides a comprehensive summary of AEs reported across the included studies. The data are categorised by various AE types. In observational studies, key AEs observed include QTcF prolongation (>500 ms), documented in 22 studies ranging from 0% to 12.12%. Hepatic disorders or elevated liver enzymes were reported in 29 studies, ranging from 0% to 31.62%. Renal failure or increased creatinine levels were noted in 20 studies, ranging from 0% to 32.43%. Optic neuropathy or blurred vision was found in 18 studies, with a range from 0% to 11.42%. Ototoxicity or hearing loss was documented in 24 studies, with a range from 4.06% to 30.76%. Haematological disorders such as anaemia, thrombocytopenia, and eosinophilia were reported in 24 studies, with a range from 3.12% to 57.69%. GI symptoms including diarrhoea, vomiting, nausea, abdominal pain, and pancreatitis were documented in 32 studies, with a range from 6.66% to 51.56%. Peripheral neuropathy was described in 26 studies, ranging from 1.02% to 20.68%. Electrolyte disturbances were reported in eight studies, with a range from 3.84% to 11.11%. Arthralgia was described in 13 studies, with a range from 2.85% to 13.16%. Psychiatric disorders were noted in 19 studies, ranging from 0.68% to 13.63%. Dermatologic symptoms were reported in 20 studies, up to 42.35%. Weakness affected patients in two studies. Cardiac disorders, specifically palpitations, were noted in 10 studies, up to 27.27%. Nervous system disorders, including memory loss and seizure, were found in 15 studies, with a range up to 11.76%. Musculoskeletal disorders, such as pain and atrophy, were documented in 16 studies, with a range from 1.56% to 21.62%. Hypothyroidism was reported in eight studies, with a range from 1.56% to 9.13%. Fever was described in one study only.^35^

The most frequently observed AEs in experimental studies were as follows: QTcF prolongation (>500 ms), described in four studies, ranging from 0 to 0.43%. GI symptoms were noted in eight studies, ranging between 5.71% and 35.71%. Hepatic disorders or elevated liver enzymes were reported in seven studies, ranging up to 40.62%. Peripheral neuropathy occurred in six studies. Ototoxicity was documented in six studies, while renal failure or increased creatinine levels were observed in four studies ranging between 0.64% and 14.28%. Haematologic AEs, reported in six studies, ranged from 1.81% to 17.85%.

### Adverse events by drug

Of the studies where injectables were used, Nguyen et al.^46^ reported hepatic (28.88%), dermatological (11.11%), haematological (6.66%), and GI (6.66%) AEs, as well as arthralgia (11.11%). In the Kang et al.^60^ study, 30 GI, 14 hepatic, 12 dermatological, and 12 ototoxicity AEs were reported. Table 1 describes AEs attributed by the physicians to specific drugs used in the treatment of MDR-TB. BDQ was linked to QT prolongation and was suspected of causing various AEs, including GI symptoms, arthralgia/myalgia, hepatotoxicity, optic neuropathy, mental/central nervous system (CNS) disorders, and diffuse hair loss.^15,37,38,47,49,52,54,60,68^ DLM was associated with QT prolongation and optic neuropathy.^47,49,60^ LZD caused polyneuropathy, QT prolongation, and hepatotoxicity. It was also suspected to cause GI symptoms, optic neuropathy, and peripheral neuropathy.^31,37,52,54^ Clofazimine was suspected to cause QT prolongation, and dermatological reactions, and was confirmed to cause optic neuropathy.^37,47,52,57,63,68^ Prothionamide is known to cause GI symptoms and hepatotoxicity and was suspected to cause CNS disorders.^52^

**Table 1.** Adverse events by drug (caused, suspected, and decreased as reported by the study’s authors).

Drug	Ototoxicity	Polyneuropathy	QT prolongation	GI symptoms	Dermatology	Arthralgia/myalgia	Hepatotoxicity	Optic	Peripheral neuropathy	Mental/CNS disorders	Diffuse hair loss	Menorrhagia	Haematologic disorders
Amikacin	Yes^31^	NA	NA	NA	NA	NA	NA	NA	Suspected^52^	NA	NA	NA	NA
Kanamycin	Suspected^13,37^	NA	NA	NA	NA	NA	NA	NA	Suspected^52^	Suspected^37^	NA	NA	NA
Capreomycin	Decreased^31^	NA	NA	NA	NA	NA	NA	NA	Suspected^52^	NA	NA	NA	NA
Linezolid	NA	Yes (*P* = 0.023)^31^	Yes^52^	Suspected^37^	NA	NA	Yes^52^	Suspected^52,54^	Suspected^37^	NA	NA	NA	Suspected^54^
Bedaquiline	NA	NA	Yes^15,37,38,47,49,52,54,60,68^	Suspected^37,52^	NA	Suspected^37^	Suspected^37,52^	Suspected^37,49^	NA	Suspected^37^	Suspected^37^	Suspected^37^	NA
Delamanid	NA	NA	Yes^47,49,60^	NA	NA	NA	NA	Yes^49^	NA	NA	NA	NA	NA
Fluoroquinolones	NA	NA	Moxifloxacin > levofloxacin^37,47,60,68^	Suspected^14^	NA	Moxifloxacin^14,37^	NA	NA	NA	NA	NA	NA	NA
Ethionamide	NA	NA	NA	Suspected^14,37^	Suspected^37^	NA	NA	NA	NA	NA	NA	NA	NA
PAS	NA	NA	NA	Suspected^37^	NA	NA	Suspected^37^	NA	NA	NA	NA	NA	NA
Clofazimine	NA	NA	Suspected^37,47,52,57,63,68^	NA	Suspected^37,47^	NA	NA	Yes^52^	NA	NA	NA	NA	NA
Pyrazinamide	NA	NA	NA	Yes^52^	Suspected^37^	Suspected^14,37,52^	Yes^52^	NA	NA	NA	NA	NA	NA
Ethambutol	NA	NA	NA	Yes^52^	NA	NA	NA	Suspected^37^	NA	NA	NA	NA	NA
Cycloserine	NA	NA	NA	NA	NA	NA	NA	NA	Suspected^37^	Suspected^13,18,37,52,54^	NA	NA	NA
Rifampicin	NA	NA	NA	NA	NA	NA	NA	NA	NA	NA	NA	Suspected^37^	NA
Aminoglycoside	Decreased^68^	NA	NA	Yes^52^	NA	Yes^52^	Yes^52^	NA	NA	NA	NA	NA	NA
Terizidone	NA	NA	NA	NA	NA	NA	NA	NA	NA	Suspected^13^	NA	NA	NA
Prothionamide	NA	NA	NA	Yes^52^	NA	NA	Yes^52^	NA	NA	Suspected^52^	NA	NA	NA

GI = gastrointestinal; CNS = central nervous system; NA = not available; PAS = para-aminosalicylic acid.

Table 2 shows a comparison of AEs associated with MDR-TB treatment described by two major individual data global studies and estimates reported in the latest WHO MDR-TB guidelines. In the article by Borisov et al.,^3^ the patients who were treated with amikacin (6.9%) and LZD (2.8%) had the highest percentage of SAEs. In the article by Lan et al.,^69^ the drugs causing the highest percentage of AEs (resulting in permanent discontinuation of a drug) were LZD (14.1%), p-aminosalicylic acid (11.6%), and amikacin (10.2%). In the WHO MDR-TB guidelines, the estimated percentage of SAEs was also highest for LZD (17.2%), p-aminosalicylic acid (14.3%), and amikacin (10.3%).

**Table 2.** Comparison of adverse events associated with MDR-TB treatment described by two major individual data global studies and estimates from the latest WHO MDR-TB guidelines.^11^

	Borisov, 2019^A^	Lan, 2020^B^	WHO MDR-TB guidelines^C^
Group A drugs			
Levofloxacin	0/241 (0%)	22/1,012 (1.3%)	4.1% (1.9–8.8)
Moxifloxacin	1/240 (0.4%)	30/904 (2.9%)	2.9% (1.4–5.6)
Bedaquiline	6/577 (1.0%)	9/464 (1.7%)	2.4% (0.7–7.6)
Linezolid	15/536 (2.8%)	140/783 (14.1%)	17.2% (10.1–27.0)
Group B drugs			
Clofazimine	3/213 (1.4%)	12/1712 (1.6%)	3.6% (1.3–8.6)
Cycloserine/terizidone	8/498 (1.8%)	337/7,547 (5.7%)	7.8% (5.8–10.9)
Group C drugs			
Ethambutol	—	124/6,089 (1.8%)	4.0% (2.4–6.8)
Delamanid	1/121 (0.8%)	—	—
Pyrazinamide	1/236 (1.7%)	410/5,151 (5.1%)	8.8% (5.6–13.2)
Imipenem/meropenem	—	9/158 (4.9%)	—
Amikacin	9/131 (6.9%)	235/4,106 (10.2%)	10.3% (6.6–17.0)
Streptomycin	—	—	4.5% (2.3–8.8)
Ethionamide/prothionamide	1/121 (0.4%)	376/4,627 (6.5%)	9.5% (6.5–14.5)
p-aminosalicylic acid	1/215 (0.5%)	532/2,929 (11.6%)	14.3% (10.1–20.7)

MDR-TB = multidrug-resistant TB.

A Serious adverse events.

B Adverse events that resulted in permanent discontinuation of a drug; pooled incidence.

C Serious adverse events; median (95% credible interval).

## DISCUSSION

Our aim was to explore the safety of treatment regimens for DR-TB as well as associated QoL over the period 2009–2024 and to identify the unmet needs. Furthermore, as part of the descriptive review, we conducted a comparative analysis of AEs associated with MDR-TB treatment described in studies based on individual patient data and WHO estimates to achieve a comprehensive understanding of the incidence and types of AEs reported across various sources.^1,3,69^

We found that the overall proportion of SAEs ranged between 0.2% and 10.1% in retrospective studies, 1.0%–72.4% in prospective cohorts, and 20.0%–25.0% in experimental studies. In studies based on individual patient data, AEs associated with BDQ (1.7%–2.4%) and FQs (3%–4%) were less frequent than those associated with LZD (14.1%–17.2%).

The variability noted was large, particularly for HIV status (from studies with no HIV-positive participants to studies in which approximately 90% of participants were HIV co-infected). Several important determinants were not evaluated in many studies/arms: BMI was reported only in 52.43%, diabetes only in 53.65%, and smoking only in 31.70% of them. The variability of the reported drug-resistance patterns was also large. The majority of studies (74%) described at least one MDR-TB patient (25% of them reporting RR-TB confirmed cases, based on Xpert MTB/RIF testing), while 35% of the studies included at least one pre-XDR-TB and 49% at least one XDR-TB affected individual. While MDR-TB, RR-TB, and XDR-TB definitions were based on the 2013 WHO classification,^70^ pre-XDR-TB was based on the 2021 WHO definition.

Due to heterogeneity of studies, it was not possible to accurately describe the compiled frequency of AEs per each individual drug. We therefore looked at various regimens prescribed and the frequency of SAEs reported for the main drugs or group of drugs likely to cause AEs. Our study confirmed that LZD is most frequently associated with toxicity: almost all the studies containing LZD reported GI and haematological AEs.

Few studies attributed AEs to a specific drug. For example, for Group A drugs, only five studies specifically attributed AEs to LZD, two to FQs, and one to BDQ; some of them reported a ‘suspected’ association only (Table 1). Therefore, a comprehensive understanding of AEs associated with individual drugs could only be achieved through analyses of international studies utilising patient-level data. Three studies were included for this. The first^3^ was a large-scale, prospective study conducted by the Global Tuberculosis Network, encompassing 45 centres across 26 countries and regions in all continents. The objective was to determine the frequency and intensity of AEs in a cohort of 658 TB patients receiving newly introduced anti-TB agents (i.e., BDQ and DLM) as well as repurposed compounds (i.e., clofazimine and LZD). The study was implemented under the framework of the WHO’s ‘active TB drug safety monitoring and management of adverse events’ (aDSM) project. Data on AEs were collected prospectively from July 2017 to August 2019, following attribution to specific drugs, together with demographic, bacteriological, radiological, and clinical variables at baseline and during treatment. This registry-based approach represented a major step towards supporting the global adoption of aDSM.

Overall, 11.1% of patients reported AEs associated with BDQ and 13.2% with DLM; however, SAEs were relatively rare, with treatment discontinuation required in only two BDQ recipients (0.35%) and one DLM recipient (0.8%), all due to cardiological complications. In comparison, 5.8% and 3.8% of patients developed AEs while receiving levofloxacin and moxifloxacin, respectively, with only two cases of SAEs reported for standard-dose moxifloxacin. The incidence of SAEs among patients treated with LZD and clofazimine was 3% and 1.4%, respectively. Notably, none of the 12 individuals treated with high-dose isoniazid or high-dose moxifloxacin experienced any AEs. Worryingly, an important proportion of AEs identified by care providers were not reported to health authorities at the national level. The authors stated that under-reporting of AEs (particularly minor ones and those not related to the new drugs) cannot be excluded. This is likely to explain the lower proportion of AEs attributed to LZD in comparison to both the Lan’s study^69^ and the WHO estimates.^1^

Lan et al.^69^ conducted a study to estimate both the absolute and relative frequencies of AEs associated with various anti-TB drugs, with the aim of providing evidence to guide clinicians and TB programmes in selecting optimal treatment regimens. The authors performed a meta-analysis based on individual-level patient data derived from studies that reported AEs resulting in permanent drug discontinuation. The dataset comprised individual records from 35 studies published between 2009 and 2016, supplemented with additional studies obtained through a WHO public call in 2018, yielding a total of 9,178 patients.

The meta-analysis of proportions indicated that drugs with comparatively low risk of discontinuation due to AEs included levofloxacin (1.3% [95% confidence interval: 0.3–5.0]), moxifloxacin (2.9% [1.6–5.0]), BDQ (1.7% [0.7–4.2]), and clofazimine (1.6% [0.5–5.3]). In contrast, higher rates of discontinuation were associated with second-line injectable agents – amikacin (10.2% [6.3–16.0]), kanamycin (7.5% [4.6–11.9]), and capreomycin (8.2% [6.3–10.7]) – as well as with aminosalicylic acid (11.6% [7.1–18.3]) and LZD (14.1% [9.9–19.6]). Among LZD-related AEs, the most frequent causes of permanent discontinuation were peripheral neuropathy (87 of 137 cases; 64%), myelosuppression (30 of 137; 22%), and optic neuritis (7 of 137; 5%).

We acknowledge that our focus on AEs that led to permanent discontinuation of the drug was a limitation; however, considering their possible impact on treatment efficacy and selection of drug-resistant mutants, we opted for reporting this finding. In addition, information on the severity or seriousness of AEs, and information on AEs that led to temporary discontinuation or dose reduction, was not available. The third source of data considered was the frequency of AEs included in the latest WHO MDR-TB guidelines.^11^ In our comparison (Table 2) among Group A drugs, there was consistency in the frequency of adverse drug reaction profile reported for BDQ, but not for FQs and LZD, the Borisov study having probably under-estimated the AEs.^3^ Within Group B, consistent results were reported for clofazimine but not for cycloserine/terizidone, again the Borisov et al.^3^ study reporting lower proportion of AEs.

In summary, the study has shown that among WHO Group A drugs (BDQ, levofloxacin, moxifloxacin, and LZD), the adverse drug reaction profile of BDQ (1.7%–2.4%) and FQs (3%–4%) is low, compared to that of LZD (14%–17%).^11^ We did not find any specific information on the toxicity of Pa, while one study reported 0.8% of SAE attributable to DLM.^3^ It is important to note the relevance of the LZD dose on AEs. Migliori and Tiberi^12^ in 2009 demonstrated for the first time that the 600 mg LZD dose was safer than the 1,200 mg one without compromising the regimen efficacy. AEs appeared after a median of 69 days (range 1–596) of LZD treatment. In total, 35 (41.2%) out of 85 patients experienced 52 episodes of AEs attributable to LZD. Only 4/28 (14.3%) receiving 600 mg of LZD experienced any side effects versus 31/57 (54.4%) who received 1,200 mg with five and 47 episodes of AEs, respectively. Of the 35 patients experiencing AEs, 27 (77.1%) experienced AEs requiring temporary or permanent discontinuation of LZD (4 in the 600 mg per day LZD usage group and 23 in the 1,200 mg LZD group), whereas 8 (22.9%) patients experienced AEs all in the regimen with 1,200 mg LZD per day. The AEs were primarily represented by anaemia (23 [44.2%] out of 52 AEs, 3 due to 600 mg LZD usage and 20 due to 1,200 mg LZD).

Among 23 patients with anaemia, five (21.7%) required blood transfusion (Hb <8 mg/dL); two had haemolytic anaemia. Other AEs included thrombocytopenia (7/52, 13.5%), nausea/vomiting (4/52, 7.7%), and polyneuropathy (3/52, 5.7%). Of 27 patients with major AEs (as defined by the authors), LZD was reintroduced in 8 (29.6%) but permanently stopped in 19 (70.4%). No LZD-related deaths occurred. AEs were significantly more common with 1,200 mg daily (31/57, 54.4%) than with 600 mg (4/28, 14.3%). Patients on 600 mg had lower risk of overall AEs. The randomised clinical trials on the BPaL/BPaLM regimen provide limited information on AEs of LZD as the primary outcome was its effectiveness.^61–63^

Not many details were provided on AEs related to specific drugs in the Nix trial.^61^ In total, 37 of 109 patients (34%) completed 26 weeks of LZD treatment without any interruption, although they may have had a dose reduction, and 16 (15%) completed 26 weeks at a total daily dose of 1,200 mg of LZD with no interruptions or dose reductions. The ZeNix trial was designed to evaluate different doses of LZD and different durations of administration.^62^ A total of 181 participants were enrolled, 88% of whom had XDR or pre-XDR-TB. Among participants who received BDQ–Pa–LZD (with LZD at a dose of 1,200 mg) for 26 weeks or 9 weeks or 600 mg for 26 weeks or 9 weeks, 93%, 89%, 91%, and 84%, respectively, had a favourable outcome. Peripheral neuropathy occurred in 38%, 24%, 24%, and 13% of patients, while myelosuppression occurred in 22%, 15%, 2%, and 7% of them, respectively. The LZD dose was modified in 51%, 30%, 13%, and 13% of patients. Optic neuropathy developed in 9% of patients on 1,200 mg for 26 weeks, but all cases resolved. Most unfavourable outcomes (6/7) occurred in the 9-week LZD groups. Overall, the best risk–benefit profile was with 600 mg LZD for 26 weeks, showing fewer AEs and dose modifications.^62^

In the PRACTECAL trial,^63^ the incidence of AEs of grade 3 or higher or SAEs was lower in the BPaLM group than in the standard-of-care group, treated with 9- to 20-month standard-care regimen (19% vs. 59%). The authors have not attributed the AEs to any specific drug.

Several studies^71–80^ evaluating safety and tolerability of MDR/RR-TB regimens were published after the search period of the present systematic review, corroborating our findings. At least three studies^71,79,80^ demonstrated that all-oral shorter regimens for MDR/RR-TB were safe and effective compared with standard therapies. In addition, BPaL/BPaLM treatment for MDR/RR-TB had favourable patient outcomes, with manageable safety profile.^76,77^ More recently, the shortened BDLC (BDQ, DLM, LZD, and clofazimine) regimen failed to meet the non-inferiority criteria when compared to the control among fluoroquinolone-resistant patients.^72^ Finally, in a randomised clinical trial,^78^ the incidence of AEs in contezolid-treated patients was lower than in LZD-treated patients, making contezolid likely a better alternative in the treatment of MDR/RR-TB.

This review has some limitations. First, there was substantial variability among studies in both the frequency and severity of AEs. Second, no relevant data on tolerability, nor QoL, were reported. Finally, a limited number of studies have clearly identified the drug responsible for each event, while the majority reported AEs at the regimen level.

## CONCLUSION

Among WHO Group A drugs, LZD, although effective, causes frequent and often severe AEs. Therefore, a systematic review of the toxicity of LZD since its introduction in anti-TB regimens will be useful, while new drugs and regimens with or without LZD are starting their track towards approval and clinical use. Patient-centred outcomes, particularly QoL, have remained largely unreported and represent a significant area for future investigation. Emerging treatment regimens, specifically those exhibiting enhanced safety profiles, could further underscore the importance of maintaining focus on tolerability and rigorous safety monitoring alongside advancements in efficacy.

## Supplementary Material
