# Supplementary material for: Safety of treatment regimens for drug-resistant TB over a 15-year period: a scoping review
Source: IJTLD Open. 2026 Apr 13;3(4):208–17. doi: 10.5588/ijtldopen.25.0728 (PMC13080302; doi:10.5588/ijtldopen.25.0728)
Supplement: Supplementary file 1 [file ijtldopen25-0728_supplementarydata1.pdf]

Table S1. Characteristics of the included studies.

Study ID	study design	country	N	regimen	duration	Mean age	male	HIV + -	BMI (Kg/m2)	diabetes status	hepatitis C	smoking	DR-TB type
Migliori 2009 [12]	RC	Multicenter	85	FQs+SLI+Lzd	7 months	NM	NM	NM	NM	NM	NM	NM	MDR-TB /XDR-TB
Brust 2013 [13]	RC	South Africa	91	Km+ Ofx+Cs+ Eto+ Z+ E+(ART regimen)	minimum of 24 months	34	41	76	NM	NM	NM	NM	MDR-TB
Hire 2014 [14]	PC	India	110	DOTS-PLUS regimen or category IV regimen: Km+ LFX+ ETO+ Z+ E+ CS (total six drugs) and continuation phase contain LFX+ETO+ E+ CS (total four drugs).	NM	>18	83	0	NM	10	NM	NM	MDR-TB
Guglielmetti 2016 [15]	RC	France	45	E+Z+Am+ Cm+ Mfx+Lfx+Eto+PAS+Cs+ Lzd+Cfz+Ipm/Cln+Mpm/Clv+BDQ	624 days	38	36	2	19.6	6	21	33	MDR-TB/XDR-TB
Ndjeka 2018 [16]	RC	South Africa	200	Bdq+Lzd+Cfz+Z+E+high dose H+ PAS+Cm+Km+Lfx+Eto+Trd	NM	34	101	134	NM	NM	NM	NM	Pre-XDR-TB/XDR
Hewison 2018 [17]	RC	Armenia(62 (75.6)) and Georgia(20 (24.4))	82	BDQ+ LZD+ IPM+AMX/CLV+CFZ+ other second line MDR-TB drugs	20-24 months	40.5	68	4	19.5	6	23	NM	MDR-TB /Pre-XDR-TB/XDR-TB
Wang 2019 [18]	RC	China	623	Standardized regimen(CS containing regimen)	24 months	42.8	447	NM	19.9	96	NM	NM	MDR-TB
Olayanju 2020 (1) [19]	PC	South Africa	82	Km + Tr d + Z + PAS + any fluoroquinilone + Mfx + Lfx + Lzd + High dose H + Eto + E + Cfz + Cm + BDQ	24months	33	50	42	NM	2	NM	NM	pre-XDR/XDR/MDR-TB
Olayanju 2020 (2) [19]	PC	South Africa	40	Km + Tr d + Z + PAS + Mpm + any fluoroquinilone + Mfx + Lfx + Lzd + High dose H + Eto + E + Cfz + Cm + BDQ + Dlm	24months	34	24	22	NM	1	NM	NM	pre-XDR/XDR/MDR-TB
Kashongwe 2020 [20]	RC	Democratique Republic of the Congo	32	Bdq+Am+Km+Lfx+Lzd+Cfz+PAS+Cs+high dose H+Z+Pto	20 months	32.4	18	3	16.9	2	NM	NM	Pre-XDR-TB/XDR
Walt 2020 (1) [21]	PC	South Africa	435	E+standardized multidrug regimen	18-24months	36.3	279	105	NM	NM	NM	NM	MDR-TB
Walt 2020 (2) [21]	PC	South Africa	278	Cs+standardized multidrug regimen	18-24months	36.3	177	66	NM	NM	NM	NM	MDR-TB
Walt 2020 (3) [21]	PC	South Africa	145	Trd+standardized multidrug regimen	18-24months	36.2	90	37	NM	NM	NM	NM	MDR-TB
Tack 2020 [22]	RC	South Africa	117	all oral,shorter regimen including BDQ and LZD	9-12 months	35	70	80	NM	NM	NM	NM	RR-TB/MDR-TB

Auchynka 2021 [23]	RC	Belarus	125	Mfx+Lfx+BDQ+Lzd+Cfz+Cs+Trd+Dlm+Z +Imp+Am+Cm+Pto+Eto+Amx/Clv	intensive phase: 6-8months + continuous phase: 12-18 months	43	90	19	22	14	19	79	MDR/XDR TB
Avaliani 2021 [24]	PC	Georgia	25	Bdq + Lzd + Lfx + Cfz + Cs+ H+ Dlm	9.2 months	48	17	2	NM	4	3	NM	RR/MDR TB
Koirala 2021 [25]	RC	Nepal	301	STR /WHO guideline	9–12 months	34	216	13	NM	28	NM	NM	MDR-TB
Govender 2023 [26]	PC	South Africa	57	BDQ+ Lfx+ Cfz+ high-dose H + E+Z (4–6 months)+ Lfx+Cfz+E+Z (5 months)	9-11 months	36	39	42	NM	NM	NM	10	MDR-TB/RR-TB
lecai 2023 [27]	RC	China	261	Z + Lfx + Mfx + Am + Cm + E + Pto + PAS + Amx/Clv + Clr + Lzd	24 months	>18	154	0	NM	9	NM	42	pre-XDR/XDR/MDR/RR-TB
Lee 2011 (1) [28]	RC	Korea	123	Lfx+ AGs+ Pto+Cs+Z+Rfm+E+PAS+ AMC+Clr+ SMX/TMP	594 days	42	69	NM	20.4	17	NM	42	MDR-TB/ XDR-TB
Lee 2011 (2) [28]	RC	Korea	48	Mfx+ AGs+ Pto+Cs+Z+Rfm+E+PAS+ AMC+Clr+ SMX/TMP	673 days	42	27	NM	19.6	5	NM	16	MDR-TB/ XDR-TB
Mpagama 2013 [29]	RC	Tanzania	61	standardized MDR-TB treatment regimen/(intensive phase:7 months)	7 months	36	41	9	NM	2	NM	16	MDR-TB
Wang 2014 (1) [30]	RC	Taiwan	44	E+H+R+Z	297.8±19.0 days	53.2	34	NM	NM	NM	NM	NM	INH-R
Wang 2014 (2) [30]	RC	Taiwan	90	E+H+R+Z	289.9±14.6 day	58.8	66	NM	NM	NM	NM	NM	INH-R
Olaru 2016 [31]	RC	Austria	90	AMC + BDQ+ CFZ +CLA+CS+ DDS +E+FQ +FUS+ IMP +INJ + LZD +PAS+PTO+Z + RFB +SXT	21 months	30	54	0	NM	NM	NM	52	XDR/MDR TB/ Pre-XDR -TB
Waghmare 2017 [32]	RC	India	194	Km/Cm + Eto + Cs + Lfx/PAS/Mfx + E + Z	24-27 months	29	NM	6	NM	22	NM	NM	MDR TB/Pre-XDR TB
Romanowski 2017 [33]	RC	Canada	165	RIF + E+ Z + FQ	10.5 months	46	103	6	NM	19	NM	NM	INH-R
Prajapati 2017 [34]	PC	India	112	intensive phase: Cm+PAS+Mfx+high dose H+ Cfz+Lzd+Amx/Clv(6-12months)+ continuation phase:PAS+Mfx+high dose H+Cfz+Lzd+Amx/Clv(18 months)	intensive phase: 6-12 months + continuous phase: 18 months (27–30 months)	33	83	1	16.79	2	NM	53	XDR-TB
Lee 2017 (1) [35]	RC	South Korea	24	E + Z + S + Cm + FQs + Pto + Cs + PAS + Lzd	18-24 months	34	13	NM	20.5	2	NM	3	MDR TB/XDR TB/ Pre-XDR -TB

Lee 2017(2) [35]	RC	South Korea	52	E + Z + S + Cm + FQs + Pto + Cs + PAS + Lzd + Dlm + BDQ + High dose H + Rfb	18-24 months	35	26	NM	20.1	5	NM	9	MDR TB/XDR TB/Pre-XDR - TB
Sarin 2019 [36]	PC	India	53	Bdq+Dlm+Imp+Mfx(High dose)+Lzd+Cfz	22 months	24	24	0	20	NM	NM	NM	pre-XDR/XDR/MDR TB
Barvaliya 2020 [37]	PC	India	127	Bdq+ Km/Cm+ Lfx/Mfx+ Eto+ Cs+ Pas+ Lzd+ Cfz+ H+Clr + Z	18 months	30.75	69	0	NM	NM	NM	NM	Pre-XDR-TB/XDR
Padayatchi 2020 (1) [38]	RC	South Africa	151	BDQ + Lzd + Mfx/Lfx + PAS + Z + Cfz + Cs/Trd + Eto + E + amx/clv + cm/km/am + H + Ipm + clr	24 weeks	33	72	116	NM	NM	NM	NM	Pre XDR TB/ XDR TB
Padayatchi 2020 (2) [38]	RC	South Africa	105	Mfx/Lfx + PAS + Z + Cfz + Cs/Trd + Eto + E + amx/clv + cm/km/am + H + clr	24 weeks	35	49	80	NM	NM	NM	NM	XDR TB
Gao 2020 [39]	RC	China	177	Z + am/Cm + Lfx/Mfx + CS + PAS + E + PTO (6 months)/ Z + Lfx/Mfx + CS + PAS + E + PTO (18 months) + background regimen	24 months	40	132	1	20	19	NM	NM	MDR TB/Pre XDR TB/ XDR TB
Vambe 2020 [40]	RC	Eswatini	352	Bdq + Dlm	NM	35	206	272	NM	NM	NM	NM	HR/MDR/XDR/Pre XDR/RR-TB
lee 2020 [41]	RC	South Korea	74	Rfb + E + Z + S + Km + Am + Lfx + Mfx + Pto + Cs + PAS + Cfz + Cpm/Clv + Lzd + BDQ + DLM	24 weeks	49.8	51	1	20.9±3.5	13	NM	NM	pre-XDR/XDR/MDR TB
kwon 2021 [42]	RC	South Korea	28	DLM+LZD+CFZ+MEM/CLV+Cs + PAS + Am + BDQ	167 days	49.5	18	0	20.2	7	NM	NM	pre-XDR/XDR TB
Sidamo 2021 (1) [43]	PC	Ethiopia	43	Mfx + H + E + Z + Pto + Cs + Cfz + LZD + BDQ + DLM + Cm + Am + Km	297 days	25	22	5	17.3	NM	NM	6	MDR-TB/Pre–XDR-TB/XDR-TB
Sidamo 2021 (2) [43]	PC	Ethiopia	37	Lfx + E + Z + Pto + Cs + Cfz + LZD + BDQ + DLM + Cm Km	522 days	26	22	6	16.7	NM	NM	4	MDR-TB/Pre–XDR-TB/XDR-TB
Hwang 2021 (1) [44]	RC	South Korea	119	BDQ containing regimen	21.1 months	51	86	0	20.5	25	NM	NM	RR-TB/MDR-TB/Pre–XDR-TB/XDR-TB
Hwang 2021 (2) [44]	RC	South Korea	141	Dlm containing regimen	21.1 months	47	96	3	21.3	19	NM	NM	RR-TB/MDR-TB/Pre–XDR-TB/XDR-TB
Sun 2021 [45]	PC	China	114	6 months intensive phase: Am + FQs + Cs + Pto + (PasiniaZid)Pa + Z / 12 months continuation phase: FQs + Cs + Pto + Pa + Z	18 months	18-65	72	0	NM	15	NM	NM	MDR-TB

Nguyen 2022 [46]	PC	Vietnam	106	BDQ + Lzd + Cfz + Lfx + Z	9-11 months	41	75	1	NM	NM	NM	NM	MDR-TB
Desai 2022 (1) [47]	PC	India	41	Bdq+OBR(optimized background regimen)	24-27 months	36.71	29	NM	16.63	NM	NM	NM	MDR-TB
Desai 2022 (2) [47]	PC	India	37	Mfx+OBR(optimized background regimen)	24-27 months	41.14	26	NM	16.48	NM	NM	NM	MDR-TB
chung 2022 [48]	RC	Korea	741	BDQ + DLM containing regimens	NM	52.1	510	4	21.1	158	NM	NM	MDR-TB
Padmapriyadarsini 2022 [49]	PC	India	165	BDQ + DLM + Pto + LZD + Cfz	24-36 weeks	27	92	0	17.3	19	NM	22	MDR-TB
Kumar G 2022 [50]	PC	India	135	intensive phase :Mfxh+Km/Am+Eto+Cfz+Z+Hh+E(4-6months)/Mfxh +Cfz+Z+E(5 months)	9-11 months	37.7	71	NM	19.8	23	NM	30	MDR-TB
Souleymane 2022 [51]	RC	South Africa	14	CM+ BDQ+ LZD+ PAS+ CFZ+ LFX+ AM+ CS+ H+ Z+ DLM	NM	NM	NM	NM	NM	NM	NM	NM	NM
Nguyen 2022 (1) [52]	PC	Vietnam	42	BDQ long + Lfx + Lzd + Cfz + Cs + Z + Pto + PAS + E	20 months	42.8	29	2	18.3	10	NM	5	Pre-XDR-TB/XDR/ MDR/RR
Nguyen 2022 (2) [52]	PC	Vietnam	57	BDQ long + Lfx + Lzd + Cfz + Cs + Z + Pto + PAS + E + High dose H + Secondline injectables + Km + Cm	20 months	44	42	1	18.4	12	NM	7	Pre-XDR-TB/XDR/ MDR/RR
Zhang 2022 (1) [53]	RC	China	102	BDQ+FQs(Lfx/Mfx)+SLI(Cm/Am)+Cs+Pto+Z+CFZ+E+LZD+PAS	18-20 months	37	78	0	NM	16	NM	NM	RR/XDR/MDR TB
Zhang 2022 (2) [53]	RC	China	100	intensive phase: FQs(Lfx/Mfx)+SLI(Cm/Am)+Cs+Pto+Z+CFZ+E+LZD+PAS	18-20 months	43.5	71	0	NM	13	NM	NM	RR/XDR/MDR TB
Prince 2023 [54]	PC	India	62	BDQ + Lzd + Cs + Mfx/Lfx + Cfz + pyridoxine	NM	37.58	44	0	NM	11	NM	NM	NM
Suvendu Kumar 2023 [55]	RC	India	95	standardised regimen	48 months	39.17	74	NM	NM	NM	NM	NM	RR/MDR-TB
Dong 2024 [56]	PC	China	143	MFX+ LZD + BDQ + Cfz + Pto + PAS + E + Cs + Z	24 months	43.3	84	0	20.9	27	0	82	MDR-TB
Mikiashvili 2024 [57]	RC	Georgia	106	BDQ and Dlm containing regimens + LZD + Cfz + Cs + FQs	479 days	39.5	79	4	20.6	15	28	67	RR/MDR-TB/Pre-XDR TB/XDR TB
Esmail 2022 (1) [58]	RCT	South Africa	49	BDQ+Lzd+Lfx+Z+Trd/Eto/high dose H	6-9months	37	34	27	NM	NM	NM	24	MDR/RR-TB
Esmail 2022 (2) [58]	RCT	South Africa	44	Km+Mfx+Cfz+Z+Trd/Eto/high dose H(18-20months)+Km+Mfx/	18-20 months+ 9-11months	36	28	24	NM	NM	NM	22	MDR/RR-TB

				Lfx+Cfz+z+E+Trd/Eto/high dose H(9-11months)									
Tang 2014 (1) [59]	RCT	China	33	Pto+Z+Mfx/Gfx/Lfx/PAS+CM+Am+CFZ+CLr+LZD	24 months	44	22	0	19.5	6	NM	NM	XDR-TB
Tang 2014 (2) [59]	RCT	China	32	Pto+Z+Mfx/Gfx/Lfx/PAS+CM+Am+CFZ+CLr	24 months	43	21	0	19.6	6	NM	NM	XDR-TB
Kang 2016 (1) [60]	RCT	South Korea	77	Lfx+Rfb+E+Z+injectable drugs(STR+Km+Am)+Pto+Cs+PAS+LZD	19.9 months	44	54	NM	19.8	1	NM	NM	MDR-TB
Kang 2016 (2) [60]	RCT	South Korea	74	Mfx+Rfb+E+Z+injectable drugs(STR+Km+Am)+Pto+Cs+PAS+LZD	19.7 months	42	48	NM	20.7	4	NM	NM	MDR-TB / XDR-TB
Conradie 2020 [61]	RCT	South Africa	109	BDQ+LZD+Pa	26 Weeks	35	57	56	19.7	NM	NM	NM	MDR-TB /XDR-TB
Conradie 2022 [62]	RCT	multicentre	181	BDQ+LZD+Pa	26 Weeks	36	122	36	20.8	9	NM	66	Pre-XDR TB/XDR TB/ RR-TB
Nyang'wa 2022 (1) [63]	RCT	multicentre	152	Standard-Care	9-to-20-month	37	96	41	19.9	NM	NM	NM	RR-TB
Nyang'wa 2022 (2) [63]	RCT	multicentre	151	BDQ+Pa+Lzd+Mfx	24 weeks	35	85	38	19.8	NM	NM	NM	RR-TB
Nyang'wa 2022 (3) [63]	RCT	multicentre	126	BDQ+Pa+Lzd+Cfz	24 weeks	32	84	33	19.5	NM	NM	NM	RR-TB
Nyang'wa 2022 (4) [63]	RCT	multicentre	123	BDQ+Pa+Lzd	24 weeks	35	65	41	20	NM	NM	NM	RR-TB
Yao 2023 (1) [64]	RCT	China	34	BDQ+LFX+LZD+CS+CFZ(6months)+LFX+LZD+CS+CFZ(12months)	18 months	43	19	0	20.3	NM	NM	NM	MDR-TB
Yao 2023 (2) [64]	RCT	China	34	BDQ+Lfx+LZD+Cs+Pto(E)(6months)+Lfx+lzd+Cs+Pto(E)(12months)	18 months	46	18	0	19.5	NM	NM	NM	MDR-TB
Diacon 2014 (1) [65]	RCT	multicentre	66	Eto+Z+Ofx+Km+Cs+Bdq	18-24 months	32	45	5	NM	NM	NM	NM	NM
Diacon 2014 (2) [65]	RCT	multicentre	66	Eto+Z+Ofx+Km+Cs+ placebo	18-24 months	34	40	14	NM	NM	NM	NM	NM
Du 2019(1) [66]	RCT	China	67	Cm+CFZ+Cs+Lfx+Pto+Z(6months)+CFZ+Cs+Lfx+Pto+Z(6months)	12 months	37.9	44	0	19.8	1	NM	NM	MDR-TB
Du 2019(2) [66]	RCT	China	68	Cm+E+Cs+Lfx+Pto+Z(6months)+E+Cs+Lfx+Pto+Z(12months)	18 months	39	45	0	20.1	3	NM	NM	MDR-TB
Duan 2019 (1) [67]	RCT	China	66	Am/Cm+Lfx+Z+E+PAS+/Pto+Amx/Clv+CFZ(24months)	24 months	36.8	44	0	19.9	2	NM	NM	MDR-TB
Duan 2019 (2) [67]	RCT	China	74	Am/Cm+Lfx+Z+E+PAS+/Pto+Amx/Clv(6months)+Lfx+Z+E+PAS/Pto+Amx/Clv(18months)	24 months	36.4	44	0	19.8	2	NM	NM	MDR-TB

Goodall 2022 (1) [68]	RCT	multicentre	196	high dose Lfx+CFZ+E+Z(40 weeks)+ BDQ+ high dose H+ Pto(16 weeks/intensive phase)	40·1 weeks	NM	124	27	NM	NM	NM	31	RR-TB
Goodall 2022 (2) [68]	RCT	multicentre	187	high dose Mfx+CFZ+E+Z(40 weeks)+ Km+ high dose H+ Pto(16 weeks/intensive phase)	40·1 weeks	NM	115	25	NM	NM	NM	28	RR-TB
Goodall 2022 (3) [68]	RCT	multicentre	134	BDQ+CFZ+Z+Lfx(28 weeks)+ high dose H+Km(8 weeks/intensive phase)	6 months	NM	81	21	NM	NM	NM	16	RR-TB
Goodall 2022 (4) [68]	RCT	multicentre	127	high dose Mfx+CFZ+E+Z(40 weeks)+ Km+ high dose H+ Pto(16 weeks/intensive phase)	40·1 weeks	NM	77	21	NM	NM	NM	22	RR-TB

RC: retrospective cohort study; PC: prospective cohort study; RCT: randomized controlled trial; BMI: body mass index; DR-TB: drug resistance tuberculosis; RR: rifampin-resistant; MDR: multidrug-resistant; XDR: extensively drug resistant; NM: not mentioned; Km: kanamycin; Ofx: ofloxacin; CS: cycloserin; Eto: ethionamide; BDQ: bedaquiline; LFX: levofloxacin; CFZ: clofazimine; Z: pyrazinamide; E: ethambutol; ART: antiretroviral therapy; Am: amikacin; Cm: capromycin; Mfx: moxifloxacin; PAS: para-aminosalicylic acid; LZD: linezolid; Ipm/Clm: Imipenem-cilastatin; Mpm/Clv: meropenem-clavulanic acid acid; H: isoniazid; Trd: terizidone; FQs: fluoroquinolones; DLM: delamanide Amx/Clv:Amoxicillin-clavulanic acid ;STR : short treatment regimen; AGs: aminoglycosides; Rfm: rifampicin; SMX/TMP: Sulfamethoxazole-trimethoprim; FUS:fusidic acid; INJ: injectable drugs; RFB: rifabutin; SXT: co-trimoxazole; RIF: rifamycin; S: streptomycin; Clr: clarithromycin; OBR: optimized background regimen; Cpm: chlorphenamine; SLI: second-line injectable drugs; PN: pyridoxine

Table 2. Total number of adverse events in the included studies.

Study ID	Study design	No. of patients	Regimen	Duration of treatments	Number of any AEs	SAE (% SAEs of AEs)
**SHORTER REGIMENS**						
Kwon 2021 [42]	RC	28	DLM+LZD+CFZ+MEM/CLV+Cs + PAS + Am + BDQ	167 days	NM	NM
Padayatchi 2020 (1) [38]	RC	151	BDQ + Lzd + Mfx/Lfx + PAS + Z + Cfz + Cs/Trd + Eto + E + amx/clv + cm/km/am + H + Ipm + clr	24 weeks	449	NM
Padayatchi 2020 (2) [38]	RC	105	Mfx/Lfx + PAS + Z + Cfz + Cs/Trd + Eto + E + amx/clv + cm/km/am + H + clr	24 weeks	NM	NM
Lee 2020 [41]	RC	74	Rfb + E + Z + S + Km + Am + Lfx + Mfx + Pto + Cs + PAS + Cfz + Cpm/Clv + Lzd + BDQ + DLM	24 weeks	NM	NM
Nyang'wa 2022 (2) [63]	RCT	151	BDQ+Pa+Lzd+Mfx	24 weeks	1559	NM
Nyang'wa 2022 (3) [63]	RCT	126	BDQ+Pa+Lzd+Cfz	24 weeks	1401	NM
Nyang'wa 2022 (4) [63]	RCT	123	BDQ+Pa+Lzd	24 weeks	1336	NM
Goodall 2022 (3) [68]	RCT	134	BDQ+CFZ+Z+Lfx(28 weeks)+ high dose H+Km(8 weeks/intensive phase)	6 months	NM	NM
Migliori 2009 [12]	RC	85	FQs+SLI+Lzd	7 months	52	NM
Mpagama 2013 [29]	RC	61	standardized MDR-TB treatment regimen/(intensive phase:7 months)	7 months	NM	NM
Conradie 2022 [62]	RCT	181	BDQ+LZD+Pa	26 Weeks	740	NM
Conradie 2020 [61]	RCT	109	BDQ+LZD+Pa	26 Weeks	NM	NM
Padmapriyadarsini 2022 [49]	PC	165	BDQ + DLM + Pto + LZD + Cfz	24-36 weeks	NM	33
Esmail 2022 (1) [58]	RCT	49	BDQ+Lzd+Lfx+Z+Trd/Eto/high dose H	6-9months	56	14 (25)
Wang 2014 (1) [30]	RC	44	E+H+R+Z	297.8±19.0 days	NM	NM
Sidamo 2021 (1) [43]	PC	43	Mfx + H + E + Z + Pto + Cs + Cfz + LZD + BDQ + DLM + Cm + Am + Km	297 days	NM	NM
Wang 2014 (2) [30]	RC	90	E+H+R+Z	289.9±14.6 day	NM	NM
Avaliani 2021 [24]	PC	25	Bdq + Lzd + Lfx + Cfz + Cs+ H+ Dlm	9.2 months	11	3(27.27)
Nguyen 2022 [46]	PC	106	BDQ + Lzd + Cfz + Lfx + Z	9-11 months	45	13(28.88)
Govender 2023 [26]	PC	57	BDQ+ Lfx+ Cfz+ high-dose H + E+Z (4–6 months)+ Lfx+Cfz+E+Z (5 months)	9-11 months	95	1(1.05)
Kumar G 2022 [50]	PC	135	intensive phase :Mfxh+Km/Am+Eto+Cfz+Z+Hh+E(4-6months)/Mfxh +Cfz+Z+E(5 months)	9-11 months	NM	NM
Goodall 2022 (1) [68]	RCT	196	high dose Lfx+CFZ+E+Z(40 weeks)+ BDQ+ high dose H+ Pto(16 weeks/intensive phase)	40·1 weeks	NM	NM
Goodall 2022 (2) [68]	RCT	187	high dose Mfx+CFZ+E+Z(40 weeks)+ Km+ high dose H+ Pto(16 weeks/intensive phase)	40·1 weeks	NM	NM
Goodall 2022 (4) [68]	RCT	127	high dose Mfx+CFZ+E+Z(40 weeks)+ Km+ high dose H+ Pto(16 weeks/intensive phase)	40·1 weeks	NM	NM
Romanowski 2017 [33]	RC	165	RIF + E+ Z + FQ	10.5 months	70	NM
Koirala 2021 [25]	RC	301	STR /WHO guideline	9–12 months	NM	55
Tack 2020 [22]	RC	117	all oral,shorter regimen including BDQ and LZD	9-12 months	298	NM
Du 2019(1) [66]	RCT	67	Cm+CFZ+Cs+Lfx+Pto+Z(6months)+CFZ+Cs+Lfx +Pto+Z(6months)	12 months	35	NM
**LONGER REGIMENS**						
Mikiashvili 2024 [57]	RC	106	BDQ and Dlm containing regimens + LZD + Cfz + Cs + FQs	479 days	NM	NM
Sidamo 2021 (2) [43]	PC	37	Lfx + E + Z + Pto + Cs + Cfz + LZD + BDQ + DLM + Cm Km	522 days	NM	NM

Barvaliya 2020 [37]	PC	127	Bdq+ Km/Cm+ Lfx/Mfx+ Eto+ Cs+ Pas+ Lzd+ Cfz+ H+Clr + Z	18 months	NM	NM
Sun 2021 [45]	PC	114	6 months intensive phase: Am + FQs + Cs + Pto + (PasiniaZid)Pa + Z / 12 months continuation phase: FQs + Cs + Pto + Pa + Z	18 months	42	NM
Yao 2023 (1) [64]	RCT	34	BDQ+LFX+LZD+CS+CFZ(6months)+LFX+LZD+CS+CFZ(12months)	18 months	NM	NM
Yao 2023 (2) [64]	RCT	34	BDQ+Lfx+LZD+Cs+Pto(E)(6months)+Lfx+lzd+Cs+Pto(E)(12months)	18 months	NM	NM
Du 2019(2) [66]	RCT	68	Cm+E+Cs+Lfx+Pto+Z(6months)+E+Cs+Lfx+Pto+Z(12months)	18 months	32	NM
Kang 2016 (1) [60]	RCT	77	Lfx+Rfb+E+Z+injectable drugs(STR+Km+Am)+Pto+Cs+PAS+LZD	19.9 months	NM	NM
Kang 2016 (2) [60]	RCT	74	Mfx+Rfb+E+Z+injectable drugs(STR+Km+Am)+Pto+Cs+PAS+LZD	19.7 months	NM	NM
Zhang 2022 (1) [53]	RC	102	BDQ+FQs(Lfx/Mfx)+SLI(Cm/Am)+Cs+Pto+Z+CFZ+E+LZD+PAS	18-20 months	37	NM
Zhang 2022 (2) [53]	RC	100	intensive phase: FQs(Lfx/Mfx)+SLI(Cm/Am)+Cs+Pto+Z+CFZ+E+LZD+PAS	18-20 months	26	NM
Kashongwe 2020 [20]	RC	32	Bdq+Am+Km+Lfx+Lzd+Cfz+PAS+Cs+high dose H+Z+Pto	20 months	123	NM
Nguyen 2022 (1) [52]	PC	42	BDQ long + Lfx + Lzd + Cfz + Cs + Z + Pto + PAS + E	20 months	NM	NM
Nguyen 2022 (2) [52]	PC	57	BDQ long + Lfx + Lzd + Cfz + Cs + Z + Pto + PAS + E + High dose H + Secondline injectables + Km + Cm	20 months	NM	NM
Lee 2011 (1) [28]	RC	123	Lfx+ AGs+ Pto+Cs+Z+Rfm+E+PAS+ AMC+Clr+ SMX/TMP	594 days	NM	NM
Nyang'wa 2022 (1) [63]	RCT	152	Standard-Care	9-to-20-month	2294	NM
Hwang 2021 (1) [44]	RC	119	BDQ containing regimen	21.1 months	NM	NM
Hwang 2021 (2) [44]	RC	141	Dlm containing regimen	21.1 months	NM	NM
Guglielmetti 2016 [15]	RC	45	E+Z+Am+ Cm+ Mfx+Lfx+Eto+PAS+Cs+ Lzd+Cfz+Ipm/Cln+Mpm/Clv+BDQ	624 days	NM	NM
Olaru 2016 [31]	RC	90	AMC + BDQ+ CFZ +CLA+CS+ DDS +E+FQ +FUS+ IMP +INJ + LZD +PAS+PTO+Z + RFB +SXT	21 months	NM	NM
Sarin 2019 [36]	PC	53	Bdq+Dlm+Imp+Mfx(High dose)+Lzd+Cfz	22 months	29	21(72.41)
Lee 2011 (2) [28]	RC	48	Mfx+ AGs+ Pto+Cs+Z+Rfm+E+PAS+ AMC+Clr+ SMX/TMP	673 days	NM	NM
Hewison 2018 [17]	RC	82	BDQ+ LZD+ IPM+AMX/CLV+CFZ+ other second line MDR-TB drugs	20-24 months	256	19(7.42)
Gao 2020 [39]	RC	177	Z + am/Cm + Lfx/Mfx + CS + PAS + E + PTO (6 months)/ Z + Lfx/Mfx + CS + PAS + E + PTO (18 months) + background regimen	24 months	NM	NM
Wang 2019 [18]	RC	623	Standardized regimen(CS containing regimen)	24 months	445	45(10.11)
Lecai 2023 [27]	RC	261	Z + Lfx + Mfx + Am + Cm + E + Pto + PAS + Amx/Clv + Clr + Lzd	24 months	585	NM
Dong 2024 [56]	PC	143	MFX+ LZD + BDQ + Cfz + Pto + PAS + E + Cs + Z	24 months	132	NM
Tang 2014 (1) [59]	RCT	33	Pto+Z+Mfx/Gfx/Lfx/PAS+CM+Am+CFZ+CLr+LZD	24 months	NM	NM
Tang 2014 (2) [59]	RCT	32	Pto+Z+Mfx/Gfx/Lfx/PAS+CM+Am+CFZ+CLr	24 months	NM	NM
Duan 2019 (1) [67]	RCT	66	Am/Cm+Lfx+Z+E+PAS+/Pto+Amx/Clv+CFZ(24 months)	24 months	30	NM
Duan 2019 (2) [67]	RCT	74	Am/Cm+Lfx+Z+E+PAS+/Pto+Amx/Clv(6months)+Lfx+Z+E+PAS/Pto+Amx/Clv(18months)	24 months	14	NM

Brust 2013 [13]	RC	91	Km+ Ofx+Cs+ Eto+ Z+ E+(ART regimen)	minimum of 24 months	676	NM
Olayanju 2020 (1) [19]	PC	82	Km + Tr d + Z + PAS + any fluoroquinilone + Mfx + Lfx + Lzd + High dose H + Eto + E + Cfz + Cm + BDQ	24months	250	NM
Olayanju 2020 (2) [19]	PC	40	Km + Tr d + Z + PAS + Mpm + any fluoroquinilone + Mfx + Lfx + Lzd + High dose H + Eto + E + Cfz + Cm + BDQ + Dlm	24months	125	NM
Walt 2020 (1) [21]	PC	435	E+standardized multidrug regimen	18-24months	NM	8*
Walt 2020 (2) [21]	PC	278	Cs+standardized multidrug regimen	18-24months	NM	16*
Walt 2020 (3) [21]	PC	145	Trd+standardized multidrug regimen	18-24months	NM	5*
Lee 2017 (1) [35]	RC	24	E + Z + S + Cm + FQs + Pto + Cs + PAS + Lzd	18-24 months	NM	NM
Lee 2017(2) [35]	RC	52	E + Z + S + Cm + FQs + Pto + Cs + PAS + Lzd + Dlm + BDQ + High dose H + Rfb	18-24 months	NM	NM
Diacon 2014 (1) [65]	RCT	66	Eto+Z+Ofx+Km+Cs+Bdq	18-24 months	NM	NM
Diacon 2014 (2) [65]	RCT	66	Eto+Z+Ofx+Km+Cs+ placebo	18-24 months	NM	NM
Waghmare 2017 [32]	RC	194	Km/Cm + Eto + Cs + Lfx/PAS/Mfx + E + Z	24-27 months	NM	NM
Desai 2022 (1) [47]	PC	41	Bdq+OBR(optimized background regimen)	24-27 months	NM	NM
Desai 2022 (2) [47]	PC	37	Mfx+OBR(optimized background regimen)	24-27 months	NM	NM
Suvendu Kumar 2023 [55]	RC	95	standardised regimen	48 months	37	NM
**MIXED REGIMENS**						
Auchynka 2021 [23]	RC	125	Mfx+Lfx+BDQ+Lzd+Cfz+Cs+Trd+Dlm+Z+Imp+ Am+Cm+Pto+Eto+Amx/Clv	intensive phase: 6-8months + continuous phase: 12-18 months	831	2(0.24)
Prajapati 2017 [34]	PC	112	intensive phase: Cm+PAS+Mfx+high dose H+ Cfz+Lzd+Amx/Clv(6-12months)+ continuation phase:PAS+Mfx+high dose H+Cfz+Lzd+Amx/Clv(18 months)	intensive phase: 6-12 months + continuous phase: 18 months (27–30 months)	85	27(31.76)
Esmail 2022 (2) [58]	RCT	44	Km+Mfx+Cfz+Z+Trd/Eto/high dose H(18-20months)+Km+Mfx/ Lfx+Cfz+z+E+Trd/Eto/high dose H(9-11months)	18-20 months+ 9-11months	55	11 (20)
**REGIMEN DURATION NOT AVAILABLE**						
Hire 2014 [14]	PC	110	DOTS-PLUS regimen or category IV regimen: Km+ LFX+ ETO+ Z+ E+ CS (total six drugs) and continuation phase contain LFX+ETO+ E+ CS (total four drugs).	NM	64	NM
Ndjeka 2018 [16]	RC	200	Bdq+Lzd+Cfz+Z+E+high dose H+ PAS+Cm+Km+Lfx+Eto+Trd	NM	603	87(14.42)
Vambe 2020 [40]	RC	352	Bdq + Dlm	NM	NM	NM
Chung 2022 [48]	RC	741	BDQ + DLM containing regimens	NM	NM	NM
Souleymane 2022 [51]	RC	14	CM+ BDQ+ LZD+ PAS+ CFZ+ LFX+ AM+ CS+ H+ Z+ DLM	NM	58	1(1.72)
Prince 2023 [54]	PC	62	BDQ + Lzd + Cs + Mfx/Lfx + Cfz + pyridoxine	NM	NM	NM

AE: any adverse event; SAE: serious adverse event; NM: not mentioned; RC: retrospective cohort study; PC: prospective cohort study; RCT: randomized controlled trial; Km: kanamycin; Ofx: ofloxacin; CS: cycloserin; Eto: ethionamide; BDQ: bedaquiline; LFX: levofloxacin; CFZ: clofazimine; Z: pyrazinamide; E: ethambutol; ART: antiretroviral therapy; Am: amikacin; Cm: capromycin; Mfx: moxifloxacin; PAS: para aminosalicylic acid; LZD: linezolid; Ipm/Clm: Imipenem-cilastatin; Mpm/Clv: meropenem-clavulanic acid acid; H: isoniazid; Trd: terizidone; FQs: fluoroquinolones; DLM: delamanide Amx/Clv:Amoxicillin-clavulanic acid ;STR : short treatment regimen; AGs: aminoglycosides; Rfm: rifampicin; SMX/TMP: Sulfamethoxazole-trimethoprim; FUS:fusidic acid; INJ: injectable drugs; RFB: rifabutin; SXT: co-trimoxazole; RIF: rifamycin; S: streptomycin; Clr: clarithromycin; OBR: optimized background regimen; Cpm: chlorphenamine; SLI: second-line injectable drugs; PN: pyridoxine

*Only SAEs were reported, based on patients' numbers rather than events numbers.

Table S3. Adverse events of the included studies.

Study ID	QTcF > 500	Hepatic disorder/ Elevated liver enzyme	Renal failure/ Increased creatinine	Optic neu-ropathy/ Blurred vision	Ototoxicity/ Hearing loss	Hematological disorders (Anemia, thrombocy-topenia, eosinophilia)	Gastrointestinal symptoms (Diarrhoea, vomiting, nausea, abdominal pain, pancreatitis)	Peripheral neuropathy	Electrolyte disturbance	Arthralgia	Psychiatric disorder	Dermatologic symptoms	weakness	Cardiac vascular disorders (palpitation, arrhythmia)	nervous system disorders (memory loss, seizure)	musculoskeletal disorders (pain, atrophy)	Hypothyroidism	Fever
Migliori 2009 [12]	NM	NM	NM	NM	NM	30(57.69)	4(7.69)	3(5.77)	NM	NM	NM	NM	NM	NM	NM	NM	NM	NM
Brust 2013 [13]	NM	43(6.36)	21(3.10)	NM	57(8.43)	29(4.28)	90(13.31)	66(9.76)	65(9.61)	39(5.76)	38(5.62)	48(7.1)	27(3.99)	NM	3(0.44)	32(4.73)	38(5.62)	NM
Hire 2014 [14]	NM	4(6.25)	3(4.68)	1(1.56)	NM	NM	33(51.56)	3(4.68)	NM	5(7.81)	5(7.81)	4(6.25)	NM	NM	2(3.125)	1(1.56)	1(1.56)	NM
Guglielmetti 2016 [15]	5	17	5	5	25	12	35	18	NM	6	10	8	NM	NM	NM	1	3	NM
Ndjeka 2018 [16]	NM	NM	NM	NM	NM	NM	NM	NM	NM	NM	NM	NM	NM	NM	NM	NM	NM	NM
Hewison 2018 [17]	17(6.6)	36(14.06)	9(3.5)	1(0.39)	11(4.29)	8(3.125)	63(24.60)	26(10.15)	NM	NM	NM	6(2.34)	NM	15(5.85)	17(6.64)	5(1.95)	NM	NM
Wang 2019 [18]	NM	117(26.29)	37(8.31)	NM	36(8.08)	NM	26(5.84)	NM	NM	NM	27(6.06)	NM	NM	NM	NM	NM	NM	NM
Olayanju 2020 (1) [19]	0	23(9.2)	17(6.8)	5(2)	41(16.4)	28(11.2)	59(23.6)	18(7.2)	NM	15(6)	3(1.2)	NM	6(2.4)	NM	12(4.8)	NM	6(2.4)	NM
Olayanju 2020 (2) [19]	0	13(10.4)	9(7.2)	3(2.4)	18(14.4)	15(12)	24(19.2)	12(9.6)	NM	5(4)	6(4.8)	NM	3(2.4)	NM	9(7.2)	NM	3(2.4)	NM
Kashongwe 2020 [20]	3(2.43)	1(0.81)	NM	4(3.25)	5(4.06)	17(13.82)	37(30.08)	15(12.19)	7(5.69)	NM	7(5.69)	16(13)	NM	NM	NM	NM	NM	NM
Walt 2020 (1) [21]	NM	NM	NM	NM	NM	NM	NM	NM	NM	NM	NM	NM	NM	NM	NM	NM	NM	NM
Walt 2020 (2) [21]	NM	NM	NM	NM	NM	NM	NM	NM	NM	NM	NM	NM	NM	NM	NM	NM	NM	NM
Walt 2020 (3) [21]	NM	NM	NM	NM	NM	NM	NM	NM	NM	NM	NM	NM	NM	NM	NM	NM	NM	NM
Tack 2020 [22]	NM	NM	NM	NM	NM	NM	NM	NM	NM	NM	NM	NM	NM	NM	NM	NM	NM	NM
Auchynka 2021 [23]	64(7.70)	NM	107(12.87)	NM	NM	106(12.8)	NM	NM	NM	NM	NM	NM	NM	113(13.59)	NM	NM	NM	NM
Avaliani 2021 [24]	NM	2(18.18)	NM	NM	NM	1(9.09)	1(9.09)	1(9.09)	NM	NM	1(9.09)	NM	NM	3(27.27)	NM	2(18.18)	NM	NM
Koirala 2021 [25]	NM	NM	NM	NM	NM	NM	NM	NM	NM	NM	NM	NM	NM	NM	NM	NM	NM	NM
Govender 2023 [26]	0(0)	NM	NM	NM	NM	NM	NM	2(2.10)	NM	NM	NM	NM	NM	NM	NM	NM	NM	NM
lecai 2023 [27]	NM	185(31.62)	86(14.70)	16(2.73)	28(4.78)	28(4.78)	106(18.11)	6(1.02)	NM	77(13.16)	4(0.68)	33(5.64)	NM	NM	NM	NM	16(2.73)	NM
Lee 2011 (1) [28]	NM	10	NM	2	10	2	33	NM	NM	NM	NM	4	NM	NM	13	12	NM	NM

Lee 2011 (2) [28]	NM	3	NM	0	7	4	17	NM	NM	NM	NM	2	NM	NM	7	1	NM	NM
Mpagama 2013 [29]	NM	NM	4	NM	NM	NM	NM	NM	NM	NM	3	NM	NM	NM	NM	NM	NM	NM
Wang 2014 (1) [30]	NM	8	NM	NM	NM	NM	NM	NM	NM	NM	NM	0	NM	NM	NM	NM	NM	NM
Wang 2014 (2) [30]	NM	8	NM	NM	NM	NM	NM	NM	NM	NM	NM	2	NM	NM	NM	NM	NM	NM
Olaru 2016 [31]	NM	44	NM	NM	31	NM	74	48	NM	NM	44	NM	NM	NM	NM	NM	NM	NM
Waghmare 2017 [32]	NM	3	1	4	18	NM	39	4	NM	14	37	4	NM	NM	NM	15	7	NM
Romanowski 2017 [33]	NM	20(28.57)	NM	8(11.42)	NM	NM	11(15.71)	NM	NM	2(2.85)	NM	12(17.14)	NM	NM	NM	8(11.45)	NM	NM
Prajapati 2017 [34]	NM	4(4.70)	NM	1(1.17)	5(5.88)	3(3.52)	13(15.29)	8(9.41)	NM	NM	NM	36(42.35)	NM	NM	10(11.76)	2(2.3)	NM	NM
Lee 2017 (1) [35]	NM	NM	NM	NM	NM	NM	NM	NM	NM	NM	NM	NM	NM	NM	NM	NM	NM	NM
Lee 2017(2) [35]	NM	NM	NM	NM	NM	3	2	NM	NM	1	NM	1	NM	NM	NM	1	NM	3
Sarin 2019 [36]	1(3.44)	NM	NM	NM	NM	NM	3(10.34)	5(17.24)	NM	NM	NM	NM	NM	1(3.44)	NM	NM	NM	NM
Barvaliya 2020 [37]	5	6	NM	5	4	NM	35	NM	NM	NM	NM	50	NM	NM	NM	9	NM	NM
Padayatchi 2020 (1) [38]	7(1.55)	6(1.33)	9(2.00)	34(7.57)	24(5.34)	70(15.59)	58(12.91)	56(12.47)	28(6.23)	25(5.56)	22(4.89)	17(3.78)	NM	2(0.44)	8(1.78)	NM	41(9.13)	NM
Padayatchi 2020 (2) [38]	NM	0	6	16	61	1	100	1	35	5	6	15	NM	0	1	NM	17	NM
Gao 2020 [39]	10	35	9	2	6	15	11	8	11	2	6	NM	NM	2	NM	NM	NM	NM
Vambe 2020 [40]	NM	NM	4	NM	61	NM	NM	NM	NM	NM	NM	NM	NM	NM	NM	NM	NM	NM
lee 2020 [41]	23	NM	NM	NM	NM	NM	NM	NM	NM	NM	NM	NM	NM	NM	NM	NM	NM	NM
kwon 2021 [42]	2	NM	NM	NM	NM	NM	NM	NM	NM	NM	NM	NM	NM	NM	NM	NM	NM	NM
Sidamo 2021 (1) [43]	NM	9	3	NM	9	7	5	3	NM	NM	3	NM	NM	NM	NM	11	NM	NM
Sidamo 2021 (2) [43]	NM	5	2	NM	1	6	2	1	NM	NM	1	NM	NM	NM	NM	11	NM	NM
Hwang 2021 (1) [44]	11	NM	NM	NM	NM	NM	7	NM	NM	NM	NM	NM	NM	1	0	NM	NM	NM
Hwang 2021 (2) [44]	12	NM	NM	NM	NM	NM	10	NM	NM	NM	NM	NM	NM	2	5	NM	NM	NM
Sun 2021 [45]	NM	NM	NM	NM	NM	NM	NM	NM	NM	NM	NM	NM	NM	NM	NM	NM	NM	NM
Nguyen 2022 [46]	4(8.88)	13(28.88)	NM	1(2.22)	NM	3(6.66)	3(6.66)	3(6.66)	5(11.11)	5(11.11)	NM	5(11.11)	NM	NM	NM	1(2.22)	NM	NM
Desai 2022 (1) [47]	7	3	NM	NM	2	3	21	10	NM	NM	0	47	NM	NM	5	6	NM	NM
Desai 2022 (2) [47]	2	2	NM	NM	9	2	23	5	NM	NM	1	24	NM	NM	9	6	NM	NM
Chung 2022 [48]	5	NM	NM	NM	NM	NM	6	NM	NM	NM	NM	NM	NM	NM	NM	NM	NM	NM
Padmapriyadarsini 2022 [49]	0	77	NM	NM	NM	85	24	69	NM	NM	NM	97	NM	3	NM	NM	NM	NM

Kumar G 2022 [50]	NM	NM	NM	NM	15	NM	NM	NM	NM	NM	NM	8	NM	NM	6	NM	1	NM
Souleymane 2022 [51]	2(3.44)	10(17.24)	4(6.89)	1(1.72)	7(12.06)	7(12.06)	13(22.4)	12(20.68)	NM	NM	NM	2(3.44)	NM	NM	NM	NM	NM	NM
Nguyen 2022 (1) [52]	22	13	0	3	0	6	3	0	8	0	0	NM	NM	NM	0	NM	NM	NM
Nguyen 2022 (2) [52]	22	14	4	3	3	4	3	1	10	1	2	NM	NM	NM	1	NM	NM	NM
Zhang 2022 (1) [53]	NM	9(24.32)	12(32.43)	NM	2(5.40)	2(5.40)	NM	3(8.10)	2(5.40)	NM	NM	NM	NM	NM	NM	NM	NM	NM
Zhang 2022 (2) [53]	NM	2(7.69)	3(11.53)	NM	8(30.76)	2(7.69)	NM	1(3.84)	1(3.84)	NM	NM	NM	NM	NM	NM	NM	NM	NM
Prince 2023 [54]	1	10	NM	2	1	2	22	9	23	7	2	5	NM	9	10	7	NM	NM
Suvendu Kumar 2023 [55]	NM	4(10.81)	3(8.10)	1(2.70)	3(8.10)	NM	7(18.91)	6(16.21)	NM	NM	2(5.40)	NM	NM	NM	2(5.40)	8(21.62)	NM	NM
Dong 2024 [56]	16(12.12)	10(7.57)	4(3.03)	NM	NM	11(8.33)	38(28.78)	21(15.90)	NM	NM	18(13.63)	NM	NM	NM	6(4.54)	NM	NM	NM
Mikiashvili 2024 [57]	3	14	2	NM	2	7	11	NM	NM	NM	NM	NM	NM	1	15	NM	NM	NM
Esmail 2022 (1) [58]	NM	NM	NM	NM	1(1.78)	10(17.85)	11(19.64)	12(21.42)	NM	NM	NM	9 (16.07)	NM	NM	8 (14.28)	11 (19.64)	4 (7.14)	NM
Esmail 2022 (2) [58]	NM	NM	NM	NM	19(34.54)	1(1.81)	6(10.90)	6 (10.90)	NM	NM	NM	11 (20)	NM	NM	8 (14.54)	11 (20)	3 (5.45)	NM
Tang 2014 (1) [59]	NM	6	NM	6	4	26	16	8	2	NM	NM	3	NM	3	NM	NM	NM	NM
Tang 2014 (2) [59]	NM	7	NM	0	5	5	3	1	2	NM	NM	3	NM	2	NM	NM	NM	NM
Kang 2016 (1) [60]	NM	14	NM	4	12	2	30	4	NM	NM	7	12	NM	0	NM	29	NM	NM
Kang 2016 (2) [60]	NM	11	NM	2	12	3	28	1	NM	NM	4	7	NM	0	NM	11	NM	NM
Conradie 2020 [61]	NM	8	4	34	10	53	73	10	6	6	10	69	NM	22	82	31	NM	NM
Conradie 2022 [62]	2(0.27)	6(3.5)	17(2.29)	15(2.02)	4(0.54)	32(4.3)	48(6.48)	45(6.08)	7(0.94)	3(0.4)	14(1.89)	53(7.16)	NM	26(3.5)	60(8.10)	17(2.29)	NM	NM
Nyang'wa 2022 (1) [63]	10(0.4)	14(0.61)	19(0.82)	12(0.52)	23(1.00)	146(6.36)	410(17.87)	28(1.22)	1(0.04)	48(2.09)	27(1.17)	163(7.10)	NM	42(1.83)	212(9.2)	62(2.7)	3(0.13)	NM
Nyang'wa 2022 (2) [63]	1(0.06)	15(0.96)	12(0.76)	21(1.34)	21(1.34)	122(7.82)	252(16.16)	14(0.89)	0(0.0)	13(0.83)	13(0.83)	76(4.87)	NM	30(1.92)	160(10.26)	40(2.56)	0(0)	NM
Nyang'wa 2022 (3) [63]	3(0.21)	10(0.71)	9(0.64)	11(0.78)	11(0.78)	80(5.7)	165(11.77)	10(0.71)	0(0.0)	7(0.49)	9(0.64)	134(9.56)	NM	32(2.28)	109(7.78)	24(1.71)	5(0.35)	NM
Nyang'wa 2022 (4) [63]	0(0)	12(0.89)	17(1.27)	8(0.59)	13(0.97)	92(6.88)	117(8.75)	16(1.19)	1(0.07)	25(1.87)	7(0.52)	107(8.00)	NM	34(2.54)	173(12.94)	34(2.54)	1(0.07)	NM
Yao 2023 (1) [64]	1	NM	NM	NM	NM	NM	NM	NM	NM	NM	NM	NM	NM	NM	NM	NM	NM	NM

Yao 2023 (2) [64]	0	NM	NM	NM	NM	NM	NM	NM	NM	NM	NM	NM	NM	NM	NM	NM	NM	NM
Diacon 2014 (1) [65]	NM	NM	NM	NM	NM	NM	NM	NM	NM	29	NM	NM	NM	NM	NM	NM	NM	NM
Diacon 2014 (2) [65]	NM	NM	NM	NM	NM	NM	NM	NM	NM	22	NM	NM	NM	NM	NM	NM	NM	NM
Du 2019(1) [66]	NM	11(31.42)	5(14.28)	NM	NM	NM	2(5.71)	NM	2(5.71)	NM	2(5.71)	7(20)*	NM	NM	NM	NM	NM	NM
Du 2019(2) [66]	NM	13(40.62)	3(9.37)	NM	NM	NM	2(6.25)	NM	4(12.5)	NM	1(3.12)	0(0)*	NM	NM	NM	NM	NM	NM
Duan 2019 (1) [67]	NM	8(26.67)	NM	NM	NM	NM	3(10)	NM	NM	NM	NM	8(26.67)*	NM	NM	NM	NM	NM	NM
Duan 2019 (2) [67]	NM	2(14.28)	NM	NM	NM	NM	5(35.71)	NM	NM	NM	NM	0(0)*	NM	NM	NM	NM	NM	NM
Goodall 2022 (1) [68]	7	NM	NM	NM	NM	NM	NM	NM	NM	NM	NM	NM	NM	NM	NM	NM	NM	NM
Goodall 2022 (2) [68]	12	NM	NM	NM	NM	NM	NM	NM	NM	NM	NM	NM	NM	NM	NM	NM	NM	NM
Goodall 2022 (3) [68]	4	NM	NM	NM	NM	NM	NM	NM	NM	NM	NM	NM	NM	NM	NM	NM	NM	NM
Goodall 2022 (4) [68]	8	NM	NM	NM	NM	NM	NM	NM	NM	NM	NM	NM	NM	NM	NM	NM	NM	NM

NM: not mentioned

*There were some other subtypes of skin AEs that cannot be added to the table due to the absence of raw data.
